# Assessing the efficacy of 3D window double screens (3D-WDS) in reducing malaria transmission in northeastern Tanzania: Study protocol for a two-arm cluster-randomised controlled trial

**DOI:** 10.1016/j.conctc.2025.101503

**Published:** 2025-06-04

**Authors:** William N. Kisinza, Subam Kathet, Victor Mwingira, Maija Meri, Frank S. Magogo, Veneranda M. Bwana, Hanna Granroth-Wilding, Pendael Machafuko, Patrick Tungu, Mikko Aalto, Tomi Hakala, Markku Honkala, Seppo Meri, Ayman Khattab

**Affiliations:** a National Institute for Medical Research, Amani Medical Research Centre, Muheza, Tanzania; b Translational Immunology Research Program and Department of Bacteriology and Immunology, Faculty of Medicine, University of Helsinki, Haartmaninkatu 8, FIN-00014, Helsinki, Finland; c Biostatistics Consulting Service, Faculty of Medicine, University of Helsinki, Tukholmankatu 8B, FIN-00014, Helsinki, Finland; d Bosaso General Hospital, Bosaso, Somalia; e Department of Materials Science, Tampere University of Technology, P.O. Box 589, 33101, Tampere, Finland; f HUSLAB Diagnostic Center, Helsinki University Central Hospital, FIN-00029, Helsinki, Finland; g Department of Nucleic Acid Research, Genetic Engineering and Biotechnology Research Institute, City of Scientific Research and Technological Applications, New Borg El-Arab City, 21934, Alexandria, Egypt

**Keywords:** Malaria, Vector control, 3D-screens, House screening, LLINs, Sustainable malaria control, Cluster randomised controlled trial, Tanzania

## Abstract

**Background:**

The rise of insecticide resistance in malaria vectors has highlighted the urgent need for alternative vector control methods that do not rely on insecticides. The 3D-Screen, an innovative window screen featuring 3D conical structures integrated into a mesh, offers a promising solution. When installed as a double-screen setup (3D-Window Double Screen, or 3D-WDS) in window openings, its unidirectional design allows mosquitoes to enter the space between the screens from either the outside or inside of the living area, effectively trapping them within the enclosure. Previous laboratory and experimental hut studies have demonstrated the high efficacy of 3D-WDS in capturing host-seeking mosquitoes. This study aims to evaluate the epidemiological, entomological, and social impacts of implementing 3D-Screens in community settings.

**Methods/design:**

A two-arm, cluster-randomised controlled trial (cRCT) was conducted to assess whether houses equipped with both 3D-WDS and long-lasting insecticidal nets (LLINs) provide enhanced protection against malaria compared to LLINs alone. Twenty hamlets across 17 villages in Muheza, Tanzania, were evaluated for malaria prevalence, vector densities, entomological inoculation rates, and insecticide resistance levels. Fourteen hamlets with similar epidemiological and entomological profiles were then randomised: seven were assigned to the intervention group (3D-WDS + LLINs), and seven served as the control group (LLINs alone). Epidemiological and entomological surveillance were conducted at 10-week intervals over a 52-week follow-up period. Ancillary social science studies were conducted to assess community perceptions of the 3D-WDS intervention, focusing on acceptability and factors influencing its sustainability. Statistical analyses will use mixed-effects models to compare the impact of 3D-WDS combined with LLINs versus LLINs alone.

**Discussion:**

The 3D-WDS has the potential to reduce malaria transmission by providing a non-insecticidal, sustainable approach to mosquito control. Findings from this trial will demonstrate its real-world effectiveness and contribute to the development of scalable, long-term strategies for malaria prevention.

**Trial registration:**

ISRCTN Registry, ISRCTN87169034.

**Trial status:**

The study was initiated in June 2019, recruitment and sampling were completed in June 2021, sample analyses, and statistical evaluations are ongoing.

## 1 Introduction

### 1.1 Background and rationale

Long-lasting insecticidal nets (LLINs) and indoor residual spraying (IRS) have played a crucial role in reducing malaria worldwide and continue to be essential interventions for countries striving to move closer to malaria elimination [1,2]. However, many malaria-stricken countries, including Tanzania, are experiencing stagnation or even a reversal of the progress made [2,3]. This can be attributed to various factors, such as the increasing resistance of malaria-carrying mosquitoes to insecticides, limited access to LLINs, their short lifespan, which means they wear out before the next distribution cycle, and poor adherence to their daily use [4]. Consequently, there is a pressing need for affordable and durable vector control methods that do not rely on insecticides. These methods should provide comprehensive protection to all household members within a given community with minimal user effort and thereby help to combat insecticide-resistant malaria vectors.

The vector control tool for evaluation in this trial is a novel Three-Dimensional Screen (3D-Screen), which is installed in window openings to form a 3D-Window Double screen setup (3D-WDS). The 3D-Screens are composed of conical structures mounted on a commercial screen mesh, with each cone featuring a 5 mm diameter perforation at the tip and a fully open base with a 5 cm diameter hole. These cones are arranged at a density of 100 cones/m^2^. In a 3D-WDS setup, the larger openings of the cones face outside the double screen setup, while the smaller tips face inside, allowing mosquitoes to penetrate the screens from the outside only. Once inside, they become trapped in the space between the two screens, unable to pass through the smaller openings facing the enclosed space. The conceptualization of the 3D-Screens and their laboratory evaluation have been described and published previously [5]. The design of the 3D-WDS setup and its efficacy in semi-field conditions has also been reported [6].

In addition to providing a physical barrier, the 3D-WDS traps mosquitoes that enter houses through window openings, reducing the number of female mosquitoes able to feed on individuals carrying malaria parasites. Moreover, mosquitoes entering houses through eaves or other openings that obtain a blood meal from an infected individual and attempt to exit through windows in the morning are also trapped by the 3D-WDS. By capturing both entering and exiting mosquitoes, the intervention helps interrupt the malaria transmission cycle.

Previous mosquito control studies in endemic areas have demonstrated that house screening, which is mainly used to keep nuisance insects away, can provide protection against malaria [7,8]. A randomised controlled trial conducted in The Gambia found that window and door screens, as well as closed eaves, reduced the prevalence of anaemia in children by half [9]. Another study tested a combination of house screening and mosquito trapping as a single tool to control mosquito populations. This intervention resulted in a significant reduction of mosquitoes in houses and trapped mosquitoes were killed [10]. It is widely accepted from various studies that preventing mosquitoes from reaching humans is an effective approach to prevent mosquito-borne illnesses. House screening, which utilizes mosquito screens on windows and doors to keep mosquitoes out, is one of the methods to achieve this. The 3D-WDS is a novel mosquito screen that functions like traditional screens by blocking mosquito entry, but what sets it apart is its ability to trap mosquitoes attempting to enter and escape the house through the windows. If widely implemented, the 3D-WDS could offer additional benefits by decreasing the number of mosquitoes indoors and blood-engorged mosquitoes from spreading in the community.

### 1.2 Objectives

The objective of the presented cRCT was to investigate whether the 3D-WDS intervention provides additional protection against malaria, beyond the current standard of universal LLIN coverage (i.e., one bed net for every two individuals). As LLINs are the primary method for controlling malaria vectors worldwide [11], this study aimed to uncover any advantages offered by the 3D-WDS intervention, when used in conjunction with existing control measures. The trial took place in the Muheza District of northeast Tanzania, an area known for its high level of insecticide resistance [[12], [13], [14]]. Its aim was to assess the effectiveness and acceptability of the intervention in a setting, where conventional insecticide-based vector control methods may be compromised due to resistance.

In rural areas with high endemicity of malaria, houses are usually equipped with eaves under the ceiling to cool down the internal spaces. Additionally, other mosquito entry points typically exist on the walls of the houses. Installation of window double screens in the windows of community houses could reduce households' exposure to infectious mosquito bites. When mosquitoes attempting to enter houses are trapped inside the window double screen, households will be protected from infectious mosquito bites. Moreover, mosquitoes which manage to enter the houses and take blood meals from individuals carrying the malaria parasite will be trapped in the window double screens while trying to escape, thus blocking malaria transmission by those trapped mosquitoes. Therefore, we propose that the window double screens could reduce malaria prevalence, anaemia in children, indoor mosquito densities and the entomological inoculation rate (EIR; mean number of sporozoite infective bites/person/year).

### 1.3 Trial design

**Fig. 1:**
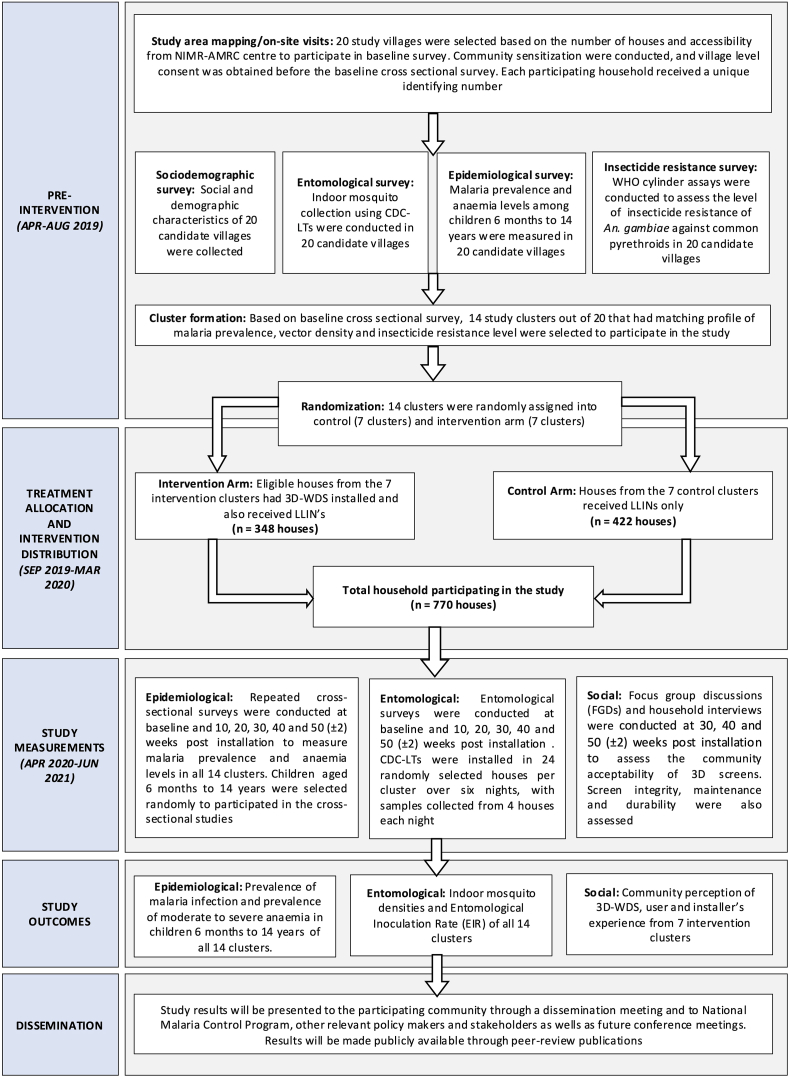
Overview of the study timeline and implementation flow for the 3D-WDS cluster-randomised trial in northeastern Tanzania.

An overview of the trial design is summarised as flowchart in Fig. 1 and the schedule of the trial activities is presented in Table 3. The study was designed as a two-arm cRCT with hamlets as the unit of randomization. The outcome measurements were conducted as repeated cross-sectional surveys of malaria and anaemia prevalence, indoor mosquito densities and entomological inoculation rate. Twenty hamlets (clusters) from 17 villages across Muheza District in the northeast of Tanzania were selected during the mapping of study area. The selection was primarily based on logistics and transport feasibility, population size and number of houses in the candidate clusters. Followed by community sensitization, a baseline survey was conducted to assess the socio-demographic profile of the candidate clusters, malaria prevalence, indoor malaria vector densities and level of insecticide resistance in candidate clusters. Fourteen clusters from the 20 candidate clusters were selected based on comparability in malaria prevalence, vector densities, and insecticide resistance profiles, to ensure balanced baseline characteristics across study arms. These 14 clusters were then randomly allocated: seven to the control arm, which received universal coverage of LLINs, and seven to the intervention arm, which received 3D-WDS installations in addition to LLINs. In both arms, existing LLINs were supplemented as needed to achieve universal coverage. Worn-out nets (e.g., those with holes) and nets estimated to be more than two years old were replaced to ensure adequate protection in all participating households. The target coverage for 3D-WDS in the intervention arm was ≥80 % of all houses in the intervention clusters. Follow-up cross sectional surveys began after the installation of intervention and LLINs rollout. Epidemiological and entomological data were collected every 10 weeks through cross-sectional surveys and ancillary social studies were conducted midway through the trial and during the final observation weeks of a 50-week follow-up period. Malariometric surveys were conducted by examining one or more children aged 6 months to 14 years living in the participating house for malaria, anaemia and general health conditions. Exposure to malaria vectors were measured using Centres for Disease Control and Prevention (CDC) miniature light traps (CDC-LTs) in both control and intervention arm. Mosquito collections were morphologically identified to species level and subjected to molecular analysis for sibling species identification, *Plasmodium* parasite and sporozoite infectivity, blood meal sources and L1014S knockdown resistance-East (*kdr*E) mutations against pyrethroids. Household and community acceptability of the 3D-WDS were assessed by organizing a series of focus group discussions (FGDs), interviews and community observation midway through the trial and during the final observation weeks of the trial. Data collections were carried out on electronic devices and stored on a secure cloud server for further processing.

## 2 Methods

### 2.1 Study setting

The trial was conducted in selected communities of Muheza District, Tanga Region of northeastern Tanzania from June 2019 to June 2021. The District covers an area of 4922 km^2^ lying between 5°10′ 0 S and 38°46′ 0 E and located at the foothills of East Usambara Mountains about 30 km offshore Indian Ocean at an elevation of 1500 m a.s.l. It borders Mkinga District to the north, to the east by the Tanga Urban and the Indian Ocean, to the south by the Pangani District, and to the west by the Handeni and Korogwe Districts. According to the 2012 Population and Housing Census, the District had a population of 204,461 with an annual population growth rate of 2.2 % with an average household size of 4.3 individuals [15]. Most of the inhabitants subsist on maize, cassava, rain-fed rice, oranges and vegetables, while some are working on sisal plantations. The climate is tropical, with dense rainforest over the Usambara mountain ranges with annual rainfall of 1000–2000 mm. The District experiences a bimodal pattern of rainfall, short rains from October to December and long rains from March to June. Malaria transmission occurs throughout the year with two seasonal peaks of mosquitoes, during and after the long and short rainy seasons of April to September and December to January [16,17]. The primary malaria vector species *Anopheles gambiae* sensu stricto*, Anopheles arabiensis* and *Anopheles funestus* are the most abundant mosquito species during the rainy season [18,19]. *An. funestus* remains common during the dry season. However, *An. gambiae s.l.* population declines as the dry season approaches. In addition to malaria vectors, other mosquitoes, such as *Culex quinquefasciatus* are abundant in the region [20]. Significant spread of resistance to pyrethroids (permethrin, deltamethrin and lambda-cyhalothrin) has been previously recorded amongst *An. gambiae* s.s population of the area [13,14,21].

### 2.2 Eligibility criteria

#### 2.2.1 Housing condition and participant eligibility

**Fig. 2:**
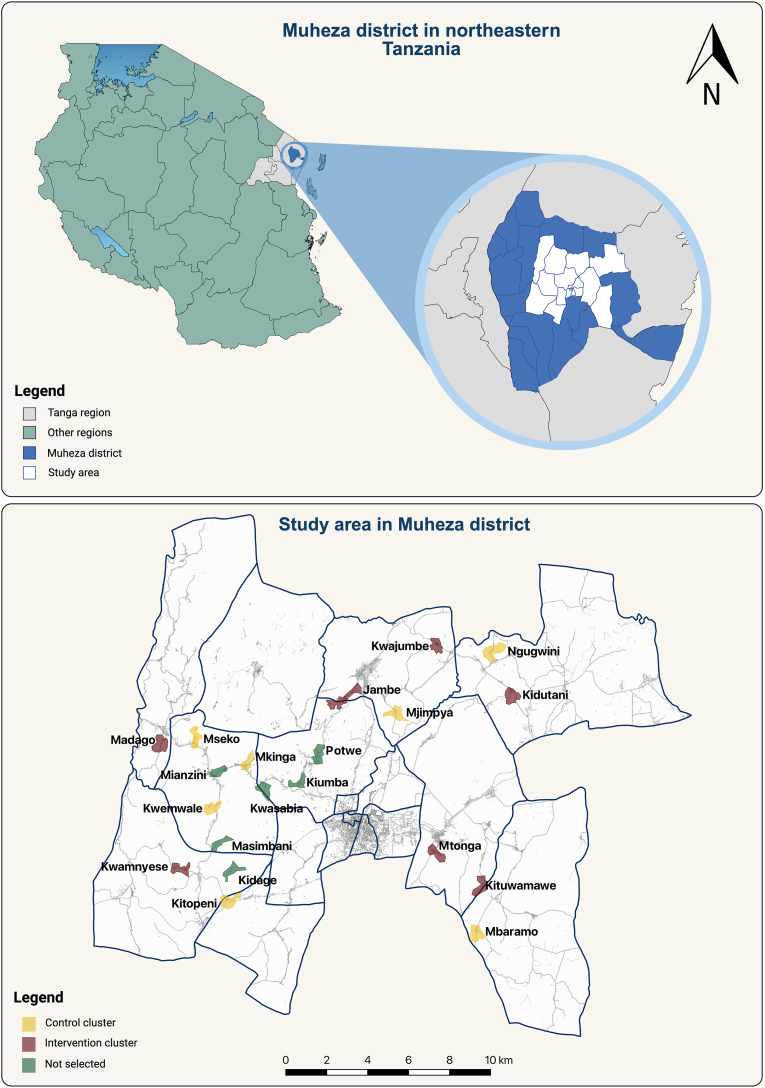
Geographic location of the study area and distribution of clusters within Muheza District, northeastern Tanzania.

The candidate clusters selected for this study were located within a 10 km radius around the town of Muheza (Fig. 2). Clusters were primarily selected based on accessibility from the project office, logistics and transportation feasibility, population size, and an average of 60–70 houses, built mostly traditionally using local resources. The selected study clusters were at least 2 km apart from any other candidate cluster to avoid potential contamination. All households with at least one child aged 6 months to 14 years old within the cluster were eligible to participate in the study. The main exclusion criteria were households without children between 6 months and 14 years and concrete houses with well-constructed windows. The detailed exclusion and inclusion criteria are outlined in Table 1.

**Table 1:** Inclusion and exclusion criteria.

SN	Condition	Inclusion criteria	Exclusion criteria
**1**	Housing	# A house constructed using traditional methods and locally sourced materials, with at least one window or opening to accommodate the 3D-WDS.	# Concrete houses with fixed window panels, shutters or well-constructed aluminium panel with glass windows.
		# Household with at least a single sleeping space.	# Spaces made for the purpose of a kitchen and dining or cattle shed.
		# Building made for long-term residential purpose (at least for the duration of the study)	# Buildings that are temporarily constructed or made for the purpose to serve as community hall, recreational centre, community mosques and churches.
		# At least one child aged 6 months to 14 years living inside the household.	# No child aged 6 months to 14 years living inside the household.
**2**	Children	# Male and female children living in the study clusters with informed parental consent.	# Children living permanently in a cluster other than the study cluster or are just visiting relatives in the study cluster.
		# Aged 6 months to 14 years.	# Aged below 6 months or above 14 years.
		# Male and female children with regular health conditions (Physical disability excluded)	# Male and female children with health conditions that require frequent hospital or health centre visits or who are allergic to antimalarial treatments.
**3**	Participation	# Consenting to the installation of the 3D-WDS within the household and the use of Insecticide-treated nets (ITNs) over a span of 12 months.	# Declining the installation of the 3D-WDS on window openings and opting not to use ITNs for a duration of 12 months.

#### 2.2.2 Implementation and ethical framework of the trial

The carpenters and technicians selected for the installation of the 3D-WDS were recruited from within the respective study cluster due to their familiarity with local house structures and locations. This local recruitment not only capitalised on their expertise and the community's trust in them but also facilitated capacity building within the trial's selected study clusters.

Experienced entomology technicians and field workers from NIMR, Amani centre, were responsible for several tasks: (i) collecting information on the feasible cost of installing the 3D-WDS, (ii) capturing mosquitoes using CDC-LTs, (iii) monitoring the installation 3D-WDS and, (iv) assessing its integrity. Additionally, trained sociologists assessed community practices and acceptance of the installed 3D-WDS in houses. Nurses/clinical officers and laboratory technicians recruited from NIMR, Amani centre, conducted clinical examination, malariometric diagnoses and took blood samples from children.

Community consent for participation in the study was obtained through sensitization meetings. Introductory letters were sent to the District medical officer of Muheza as well as to the respective village leaders, sub-leaders, and hamlet leaders. Sensitization meetings were held with village leaders, elders, community health workers and residents of the selected villages. These meetings introduced the 3D-WDS intervention, explained the concept of mosquito trapping to reduce malaria transmission, outlined the study's overall objectives, and highlighted the form participation. Additionally, the meetings covered epidemiological and entomological procedures, the study timeline, and other practical aspects. Each session concluded with a question-and-answer session where participants' queries were addressed. All meetings were conducted in the local language, Kiswahili.

All households in the intervention clusters were first assessed for eligibility based on predefined inclusion criteria, the main one being the presence of at least one child aged 6 months to 14 years. Additional considerations, such as the structural suitability of the house for 3D-WDS installation, were also applied. Households meeting these criteria were approached for consent to participate in the study, which included both the installation of the 3D-WDS and the screening of eligible children for malaria. Only households that provided informed consent were registered and assigned a unique household identification number. Refusals were rare—only two households declined participation across the seven intervention clusters.

Household registrations were conducted during the sensitization meetings, and each participating house was marked with its unique ID number. The same registered households were approached during each survey round for epidemiological follow-up. From each household, a child aged 6 months to 14 years was enrolled. If the previously screened child was unavailable, another eligible child from the same household was examined. This approach ensured consistency in sampling at the household level while allowing flexibility at the individual level.

A clinical team, comprising a clinical officer, nurses, laboratory technicians, a social scientist, and data entry personnel, handled the epidemiological data collection. Written and verbal consent was obtained from the household head or the child's caretaker in participating households prior to data collection. Verbal consent was obtained during household visits by the social scientist and nurses, who explained the clinical procedures, blood sample collection for malaria diagnostics, treatment procedures, and the frequency of clinical screenings in Kiswahili. Written consent was obtained using consent forms that outlined the study's purpose, clinical procedures, and sample collection details. Additionally, children over the age of 8 years were asked to provide assent. The social scientist addressed any questions or concerns from the households regarding the study. Epidemiological screening was performed in accordance with Tanzanian national guidelines for malaria treatment, following standard operating procedures. A trained clinical officer provided necessary treatment and dispensed medication to children with positive mRDT results. Children under two years of age with infections other than malaria were referred to designated District hospital (Teule hospital) in Muheza town using a referral form.

Entomological data collection was conducted using CDC-LTs by trained field technicians, who also obtained verbal informed consent from residents at their homes. The homes where CDC-LTs were installed were randomly selected for each round of data collection. The procedures, purpose, and frequency of the collection were explained to the head of each household.

Consent forms, written in Kiswahili, included logos, the study title, and the contact details of the involved institutions. Participants were informed of their right to withdraw from the study at any time during the sensitization meetings and informed consent process. Forms were read by the team member before signing and were signed by both parties: the household head or adult guardian of the participating household and an investigator or a representative from the institute. All consent forms were duly registered and documented for future reference.

Blood samples collected on filter papers as blood spots will be stored anonymously in the laboratory of Department of Bacteriology and Immunology at the University of Helsinki for five years and will be used for ancillary studies. Consent for the use and storage of blood samples will be obtained during the participant enrolment. Study participants may voluntarily opt out on them without refusal in the trial.

### 2.3 Interventions

#### 2.3.1 Control arm: standard pyrethroid LLINs

According to the World Health Organization (WHO) Vector Control Advisory Group's (VCAG) guidelines for field trials on vector control, it is essential for studies to include a control group running concurrently with the intervention arm [22]. This allows for simultaneous data collection from both arms. In this cluster-randomised controlled trial (cRCT), the control arm received the core prevention tool against malaria recommended by WHO: pyrethroid-treated LLINs, rather than the 3D-WDS intervention. No modifications or screenings were performed on houses in the control arm. All LLINs distributed in the study were WHO-approved blue rectangular Interceptor® nets (160 cm wide x 180 cm long x 180 cm high), coated with alphacypermethrin (200 mg/m^2^) on polyester fibers [23]. These were pyrethroid-only LLINs, consistent with the nets already present in the study area. To ensure homogeneity across households, the same type of LLINs was provided when supplementing existing nets to achieve universal coverage. Participants were not instructed to refrain from using other vector control methods such as coils, topicals or additional insecticide-treated nets.

#### 2.3.2 Intervention arm: 3D-WDS + LLINs

**Fig. 3:**
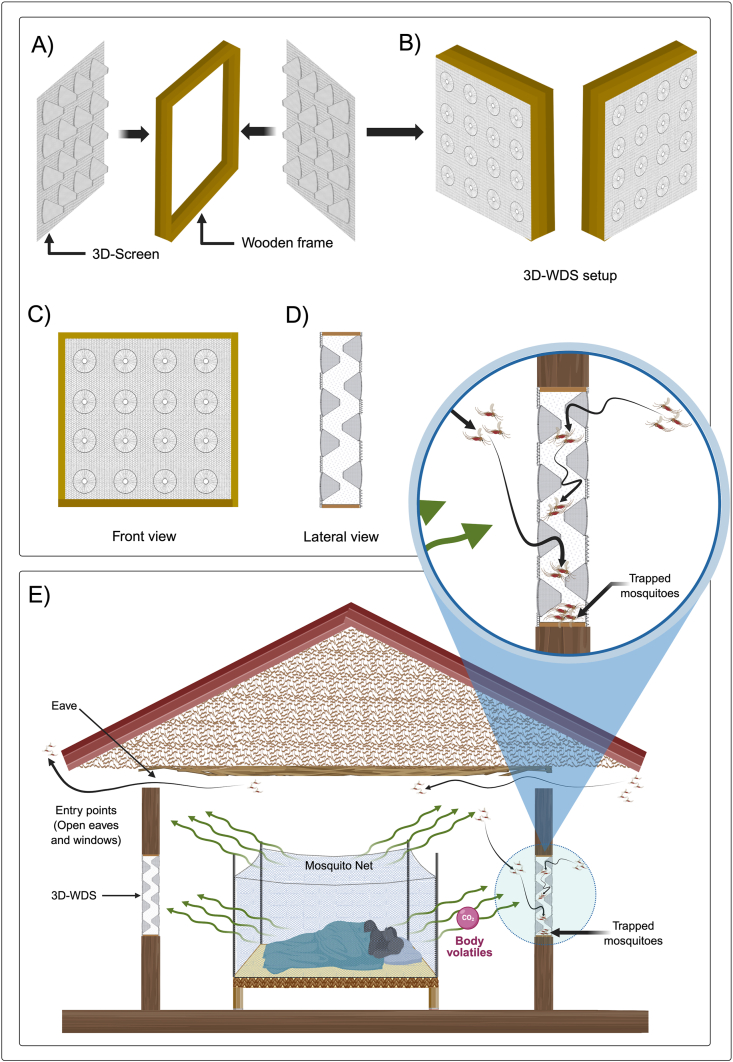
(A) Assembly process showing two 3D-Screens and a wooden frame. (B) Final configuration of the 3D-WDS setup, with screens mounted on either side of the frame. (C) Front view of a completed 3D-WDS unit. (D) Lateral view showing the conical structure of the screen material. (E) Application on a typical mud house. Mosquitoes are attracted to host odors and CO_2_ from inside the house and enter through open eaves and windows. Those entering through windows are trapped between the screens. Those entering through eaves and attempting to exit via windows after feeding are also trapped, thereby reducing malaria transmission potential.

**Fig. 4:**
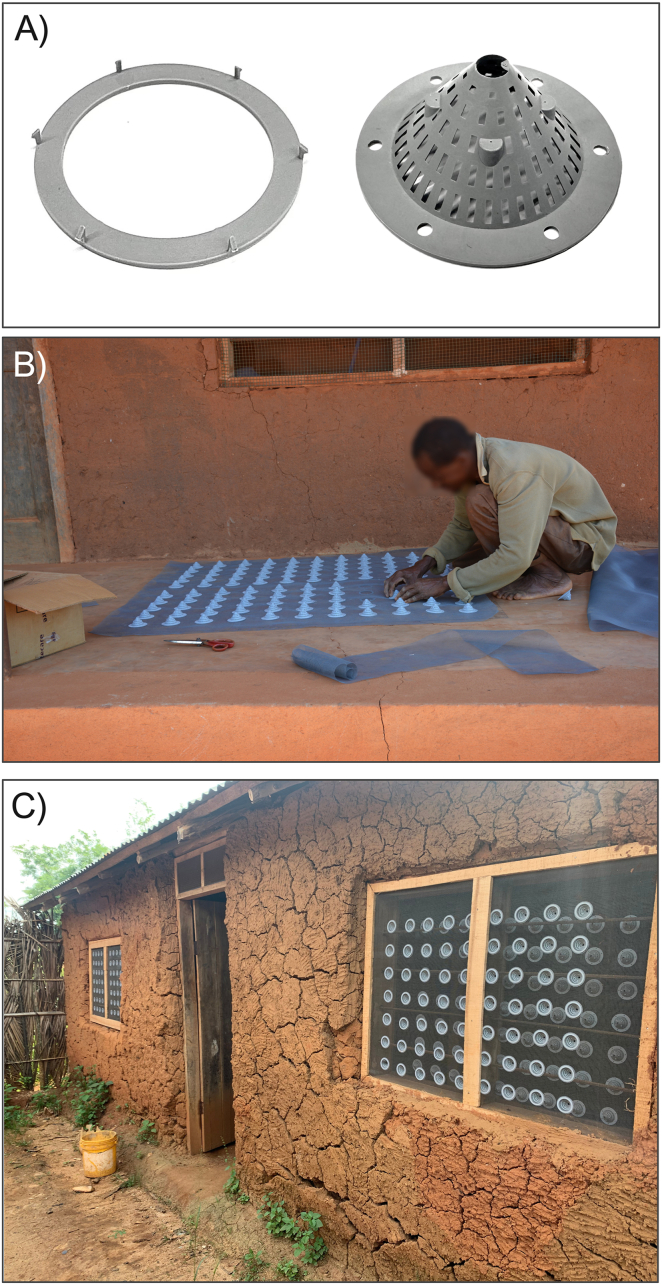
Real-world components and installation of 3D-WDS in the study area. (A) Injection-molded plastic cone (right) and its corresponding mounting ring (left), which includes integrated hooks for fastening the cone onto the mesh screen. (B) A trained community carpenter assembling the 3D-WDS by attaching cones to the mesh using mounting rings. (C) Fully installed 3D-WDS units on the window frames of a traditional mud-plastered house in the study area.

Houses in the intervention arm received 3D-WDS installations on the window openings of all sleeping spaces, in addition to receiving the core malaria prevention tool LLINs. The 3D-WDS were applied only to window openings; doors and eaves were not covered by the intervention. Window openings in cooking areas and cattle sheds were not offered 3D-WDS. Some houses also received some modifications on window openings such as reinforced wooden frames, increased window sizes and new plaster lining. A diagram illustrating the 3D-WDS installation on wooden window frames and a screened house is shown in Fig. 3. Fig. 4 shows the real-world application of the 3D-WDS during field implementation, including the preparation of the screen units and their installation on typical mud-plastered houses in the study area. All expenses incurring from installation and house modification activities were covered by the project. The majority of households in the rural areas of the Muheza District are constructed using mud and wood and/or plastered with mud. The installation exercise excluded some houses built with bricks and without eaves, concrete houses with iron roofing and well-constructed (using aluminium panel and glass) windows. Community churches, mosques, schools, community hall and houses built for purposes other than residence were also excluded. Most mud houses did not appear to have proper windows and had non-screened openings only, with textile fabrics cover. These window-like openings were also screened with the 3D-WDS. Regular inspection and monitoring of the installed 3D-WDS took place every 10 weeks within the lifespan of the trial to identify and repair any damages. House owners were also given instructions on cleaning and maintaining the 3D-WDS and reporting damage and maintenance issues to the study field workers.

### 2.4 Participant withdrawal and ethical considerations

The study ensured that participants had the freedom to withdraw from the study at any point, without providing an explanation. In the event that the homeowners requested their house to be restored to the pre-intervention state, any 3D-WDS installed were taken down and replaced with the original structure. Withdrawals could also occur, if a child from the participating household had any clinical abnormalities, intercurrent illness, or other medical condition or situation that occurred so that continued participation in the study was not in the best interest of the participant. If at least one child was not consistently available for the cross-sectional surveys in a household, that household became ineligible for participation, and the related data was excluded from the analyses. If a household withdrew consent, no further follow-up was made in that household. If this household was participating in the entomology collections (done in a randomly selected subset of households; details below), it was replaced by a neighbouring participant household. The study team made sure that they respected the participants' rights and ensured that there were contingency plans in place for any unforeseen events that might compromise the study's validity.

### 2.5 Monitoring and maintenance of 3D-WDS installations

The distribution of 3D-WDS was followed by monitoring and observation of the fully installed window screens at 10 weeks intervals. The fibre-mesh and cone structures of the 3D-WDS were inspected for any damage, and surveys were conducted to record data on their conditions at the time of inspection. The wooden frame of the windows was also examined, and the fit of the window within the wall opening was checked. The houses underwent inspections, and household members were questioned about the use of curtains or other materials to close windows, known reasons for any damage and whether attempts were made to fix any damage. The trial staff repaired any damage to the fibre-mesh, or the cone structures observed during the inspections as well as during the entomological and epidemiological surveys.

### 2.6 Integration of standard malaria control practices

The standard practice of clinical management of malaria and the management of mosquitoes within the community (such as LLINs and IRS) were not withheld in either study group. However, these practices were documented during the baseline demographic survey and household surveys conducted post-intervention. Children who tested positive in malaria rapid diagnostic tests (mRDT) tests during both the baseline and follow-up were administered a 3-day course of **artemether-lumefantrine (ALu) (Coartem®)**. Additionally, participants were encouraged to continue using LLINs and were not instructed to refrain from using alternative vector control methods (such as coils, sprays and repellents). This approach allowed for an assessment of the impact of using 3D-WDS, while assuming that all other vector control measures remained in place for malaria prevention.

### 2.7 Outcomes

The primary and secondary outcome measures of this study are outlined in Table 2.

**Table 2:** Study outcome, analysis, and collection method.

Outcome	Method	Frequency
**Primary outcome**		
Malaria prevalence	mRDT	Cross-sectional surveys at baseline and 10, 20, 30, 40 and 50 (±2) weeks post installation
**Secondary outcomes**		
Anaemia	Hemocue analyser	same as above
Indoor mosquito densities	CDC-LTs	same as above
Entomological inoculation rate	RT-PCR/CDC-LTs	same as above
Community perception and acceptability	FGD's and interviews	Cross-sectional surveys at 30, 40 and 50 (±2) weeks post installation

**Table 3:** Study timeline.

	Apr–May 2019	June–Aug 2019	Sep 2019–Mar 2020	Apr 2020–Jun 2021	Jul–Oct 2021
1. Preparatory Phase					
Study area mapping/on-site visits	X				
Candidate village selection	X				
Community engagement/Sensitization	X				
Enrolment and Informed consent	X	X			

2. Baseline surveys					
Sociodemographic survey		X			
Entomological survey		X			
Malariometric survey		X			
Insecticide resistance survey		X			

3. Enrolment					
Cluster selection for cRCT			X		
Workshop training for carpenters and technicians			X		
Installation of the 3D-WDS			X		
Distribution of LLINs			X		

4. Follow-up surveys					
Epidemiological				X	
Entomological				X	
Community perception and acceptance				X	
Intervention monitoring and repairs				X	

5. Close-out					X

### 2.8 Participant timeline

The study spanned 2 years and 7 months (April 2019 to October 2021). The initial 6 months were dedicated to site selection, community engagement and the baseline survey. An additional 7 months were allocated for cluster formation and randomization, training of installers, installation of the 3D-WDS and distribution of LLINs. The cRCT itself lasted approximately 12 months, followed by a 3-month close-out stage.

The baseline survey began in May 2019, alongside community sensitization and a sociodemographic survey. Baseline entomological and epidemiological surveys were conducted in parallel during June and July 2019. Insecticide resistance surveys were carried out shortly after the long rainy season, between July and August 2019.

Cluster formation and randomization occurred in late August 2019, followed by the recruitment of installers and workshop training for 3D-WDS installation in September 2019. The installation process took 5 months (October 2019 to February 2020), and LLINs distribution occurred in early March 2020.

Epidemiological and entomological follow-up assessments were conducted in parallel through repeated cross-sectional surveys from April–May 2020 to June–July 2021, at intervals of 10, 20, 30, 40 and 50 (±2) weeks post-intervention. The epidemiological team visited one cluster per day during each follow-up survey (14 days for 14 clusters), examining children for malaria infection and anaemia. Entomological surveys involved randomly selecting 24 houses per cluster over six nights, with samples collected from four houses each night.

Ancillary social studies were conducted through FGDs and household interviews at 30, 40, and 50 (±2) weeks post-installation to assess the community's acceptance of the 3D-WDS. Screen integrity, maintenance and durability were also evaluated during these surveys.

The schedule of pre-intervention activities, baseline cross-sectional and post-intervention follow-up surveys are outlined in the table below (Table 3).

### 2.9 Sample size

The sample size calculation for the trial focused on the primary epidemiological outcome: malaria prevalence. The calculation aimed to detect a 50 % reduction in prevalence—from 44 % in the control arm to 22 % in the intervention arm—assuming a significance level of 0.05, 80 % power, and a coefficient of variation (CV) of 0.3. The power calculation was conducted using the clustersampsi command in Stata, which is based on the formula by Bennett and Hayes for cluster randomised controlled trials [24] applies a two-sample comparison of proportions. A cluster size of 50 individuals was specified in the command based on the expected number of eligible households per cluster, derived from the demographic mapping of the 20 candidate clusters.

These parameters indicated that six clusters per arm, each with approximately 50 individuals, would be sufficient to achieve the desired statistical power. To account for expected variation in cluster size (ranging from 34 to 79 households) and to safeguard against loss of power due to heterogeneity or unforeseen issues during implementation, we increased the number of clusters to 7 per arm. The average number of households per cluster remained close to the target of 50, with 422 households in the control arm and 348 in the intervention arm.

Prior to randomization, the six clusters with the greatest variance in malaria prevalence, out of the 20 included in the baseline survey, were excluded to improve baseline comparability. The remaining 14 clusters were then randomly allocated to the intervention and control arms, with seven clusters assigned to each group.

As for the study's secondary outcomes, indoor mosquito densities and Entomological Inoculation Rate (EIR), 24-night CDC-LTs collections per cluster per survey were planned for each study arm (7 clusters per arm), totaling 168-night CDC-LTs collections per arm per survey. With the average nightly indoor *Anopheles* mosquito density estimated from the CDC-LTs collections during the baseline survey at 3.2 mosquitoes per night and a coefficient of variation of 0.5, the entomological component of the study was designed to detect a 63 % decrease in indoor mosquito densities with a significance level of 0.05 and 80 % power.

The effect size assumptions used in the sample size calculation, namely a 50 % reduction in malaria prevalence and a 63 % reduction in vector density, were informed by semi-field experimental hut trials conducted in Muheza, Tanzania [6]. In these trials, 3D-WDS installations captured up to 70.1 % of mosquitoes entering huts, with specific configurations (3D-WDS combined with open eaves) capturing as much as 51.2 % of female *Anopheles* mosquitoes. Although malaria prevalence was not measured directly, reductions in indoor mosquito densities are generally associated with lower malaria transmission risk. Therefore, the assumed 50 % reduction in prevalence represents a plausible extrapolation from the entomological data, based on the expected relationship between vector exposure and infection. This estimate was used as an upper-bound effect size, acknowledging that the actual impact may be more modest under field conditions.

### 2.10 Social sciences

A purposive sampling approach was applied for qualitative interviews and FGDs conducted with communities from intervention clusters. Additionally, 50 % of houses were randomly selected from those with 3D-WDS installations across the intervention clusters for structured interviews with the household heads/owners.

### 2.11 Recruitment

Community consent for participation in the study was secured through a series of sensitization meetings with village leaders, elders, community health workers, and residents. Introductory letters were sent to the District medical officer of Muheza, and to village leaders, sub-leaders, and hamlet leaders. These meetings introduced the 3D-WDS intervention, explained the concept of mosquito trapping to reduce malaria transmission, outlined the study's objectives, and highlighted the benefits of participation. The meetings also covered epidemiological and entomological procedures, the study timeline, and other practical aspects. Each meeting concluded with a question-and-answer session to address participants' queries, and all were conducted in Kiswahili.

Local carpenters and technicians from the trial villages were recruited for the 3D-WDS installation to enhance community engagement and participation. Following the sensitization meetings, household registration and enumeration were conducted, and a baseline survey and cluster formation followed. Written and verbal consent was obtained from participants prior to epidemiological data collection. During the demographic study, information on housing conditions, house type, GPS location, number of sleeping spaces, and presence of children was collected to determine the number of LLINs and 3D-WDS units required.

Houses in the intervention group that were ineligible for the study received LLINs, and the reason for exclusion from 3D-WDS installation was explained to the household head. From each participating household, a child aged 6 months to 14 years was enrolled in the follow-up cross-sectional survey. If the child was absent, another child from the same household was recruited. Each participating household was assigned a unique identification number, painted in white on the main door, and participating children were also given unique identification numbers for use during the cross-sectional follow-up surveys.

### 2.12 Cluster selection and randomization

Clusters were defined as hamlets belonging to villages that comprised more than one, often distantly located, hamlet. During the mapping of the study area, 20 hamlets (clusters) from 17 villages across Muheza District in northeastern Tanzania were identified. These clusters were situated within a 10-km radius of the town of Muheza and were primarily chosen based on their accessibility from the project office, logistical and transportation feasibility, and the presence of houses constructed predominantly using traditional methods and local materials.

The number of houses per cluster ranged from 25 to 107, with a median of 53.5 houses. The mean number of houses was approximately 59.1, with a standard deviation of 20.1. Most clusters had between 40 and 70 houses, with a few outliers at either end of the distribution.

Selection of clusters for the trial was based on the baseline cross-sectional survey conducted to assess malaria prevalence, indoor malaria vector densities, and levels of insecticide resistance across the 20 candidate clusters (Fig. 2). After baseline data analysis, 14 clusters were selected for participation based primarily on malaria prevalence, while also considering vector density and insecticide resistance profiles. A strong positive correlation was observed between the density of female *Anopheles gambiae* and malaria prevalence; therefore, malaria prevalence was prioritized in cluster selection. Clusters falling within one standard deviation of the overall malaria prevalence distribution were included.

These 14 clusters were subsequently randomised into two study arms: seven clusters were allocated to the control arm and seven clusters to the intervention arm for a two-arm cluster randomised controlled trial (cRCT). Randomization was performed in R (version 3.5.2) using the randomizer package (version 1.4.2) [25].

Following randomization, the control clusters had an average of 60.3 households per cluster (range: 40–78), totaling 422 households across the group. The intervention clusters had a slightly lower average of 49.7 households per cluster (range: 34–79), totaling 348 households. This slight discrepancy in household numbers between the two arms was due to eligibility criteria applied specifically to intervention clusters, which did not apply to control clusters. Despite this difference, the number of households remained sufficient and well-distributed for robust comparisons of outcomes between arms.

As randomization was conducted without geographic stratification, the spatial distribution of clusters was not explicitly balanced regarding environmental characteristics such as elevation, land use, or proximity to water bodies. To assess potential geographic imbalances that could influence malaria transmission, we compared key physical and environmental variables across study arms.

The average elevation of clusters was similar between groups: 210.7 m in control clusters and 208.6 m in intervention clusters. Baseline larval habitat mapping also revealed broadly comparable environmental conditions. The most commonly identified larval habitats across both arms were rice plots and swamps, while dominant land use types included pasture and farmland. Vegetation coverage was generally low across all clusters. Accessibility to health services was also comparable, with mean distances and walking times to the nearest health centre being nearly identical between arms.

Although these geographic variables are not central to the intervention itself, they may influence malaria transmission risk and will therefore be included as covariates in the statistical analysis. In the planned mixed-effects models using the lme4 package in R, geographic variables that remain constant within clusters (e.g., elevation, land use type) will be treated as fixed effects, while clusters will be modelled as random intercepts to account for intra-cluster correlation.

### 2.13 Data collection and management

#### 2.13.1 Epidemiological assessments

The baseline clinical survey was conducted in all 20 candidate clusters during June/July 2019. The main objective of this cross-sectional survey was to determine malaria prevalence and anaemia amongst children aged 6 months to 14 years living in the candidate clusters. The baseline cross-sectional survey was initiated by forming a clinical screening team which comprised a study supervisor, sociologist, clinical officer, nurses, laboratory technicians and a data entry officer. Each screening started with documentation of consent and assent forms by care takers and children above 8 years of age, respectively, by the sociologist. Anthropometric measurements were taken. They included height, weight and mid upper arm circumference (MUAC) (for children under 5 years) followed by measurement of axillary temperature. Malaria infection was diagnosed subsequently using mRDT on blood obtained through finger prick sampling. Anaemia was diagnosed by measuring haemoglobin concentration using a portable β-hemoglobin HemoCue® Hb 201+ photometer (Hemocue®, Ängelholm, Sweden). Finally, blood samples were collected through the same finger prick from all consenting and participating children using Dried blood spot (DBS) technique on a filter paper following WHO guidelines [26] for parasite detection by molecular analysis. For this, a grade 3 Whatman filter paper was cut into a strip of 15 cm × 2.54 cm. Blood samples were collected on one half of the strip, dried and folded. A sticker with information of participant was placed on each DBS card. After each card was fully dried, it was numbered and packaged in a Zip bag with a silica bag enclosed to avoid moisture contamination and stored in −20 °C for subsequent analysis.

Malaria prevalence as a primary outcome of the trial was determined through repeated cross-sectional surveys in 14 study clusters every 10 weeks during the 50-week follow-up period following the procedure described above for the baseline survey. In addition to mRDT, children were also tested for malaria parasitaemia using microscopy. Thick and thin blood smears were obtained to verify the presence of *P. falciparum* and to calculate the parasite density. Thick and thin blood films were prepared, dried, and stained with Giemsa stain, and examined according to WHO guidelines [26,27]. DBS were also collected during the follow-up surveys. Children with malaria positive tests were treated with a standard 3-day course of artemether-lumefantrine (ALu) (Coartem®), an artemisinin combination therapy according to national malaria treatment guidelines set by the National Malaria Control Program (NMCP), Ministry of Health, Tanzania [11,27]. The entire procedure was administered by study nurses and clinical officer, who also gave instructions to the children's caretakers on how to take ALu emphasizing the correct timing for taking ALu tablets and the need to take the drug with a meal**.** During the trial period, the clinical team also provided treatment for non-malaria illnesses, including paracetamol if axillary temperature was ≥37.5 °C, amoxicillin for non-severe pneumonia, oral rehydration salts (ORS) plus zinc for gastroenteritis and ferrous sulphate plus albendazole for anaemia (haemoglobin <9 g/dl). Children below 2 years and those presenting with severe illness at any time were referred to Teule District hospital in Muheza town for further management. Data collection was carried out by data entry officer through pre-devised digital forms using Open Data Kit application (ODK, https://opendatakit.org) in Android tablets.

#### 2.13.2 Entomological assessments

Mosquito sampling was carried from all 20 candidate clusters during the baseline and from the 14 study clusters during the follow-up period. A total of 5 cross-sectional surveys were conducted, one every 10 weeks during the 50-week follow-up period. Indoor mosquitoes were collected using CDC-LTs installed in the sleeping spaces of houses by positioning them 50 cm away from the sleepers, who were protected with LLIN. Indoor collections were conducted from 24 randomly selected houses per cluster over six nights, with samples collected from 4 houses each night. CDC-LTs were set in the sleeping area at 18:00 p.m. and removed the following morning at 08:00 a.m.

Trapped mosquitoes were retrieved in the morning and transferred to the sorting facility, where they were identified to species level using standard morphological keys under a microscope [28]. The numbers of *An. gambiae*, *An. funestus* and other anophelines and culicines in the collection were recorded. Gonotrophic status was recorded for all female anophelines, while all males and culicines were discarded. All identified female *An. gambiae* and *An. funestus* mosquito head and thorax carcasses were further subjected to PCR analysis to detect the presence of *Plasmodium falciparum* sporozoites using a melt curve PCR assay to estimate the EIR [29]. A subset (50 %) of morphologically identified female specimens from each CDC-LTs collection were randomly selected for sibling species identification using multiplex PCR [30,31]. In addition, visibly engorged specimens were further subjected to multiplex PCR to determine the sources of blood meal using multiplex PCR method described previously [32].

#### 2.13.3 Insecticide resistance assessment

Insecticide resistance survey was conducted during the baseline surveys only. Breeding sites were mapped from the 20 candidate clusters from May–July 2019. Larvae were collected from multiple breeding sites from candidate clusters and reared to adults in the insectary of Amani centre, Muheza, NIMR. Resistance assays were carried out using WHO cylinder test for adult mosquitoes with impregnated papers obtained from the WHO supplier at Universiti Sains Malaysia. Impregnated papers were stored at 4 °C and used no more than twice and two common pyrethroids were tested at diagnostic doses (permethrin 0.75 % and deltamethrin 0.05 %) as defined by the WHO [33]. Two to five-days old female *An. gambiae* s.l. mosquitoes were tested using WHO cylinder test kit with four replicates of 15–25 wild adult female mosquitoes per cylinder. During the exposure period, knock-down (KD) rates were recorded after exposure times of 10, 15, 20, 30, 40, 50 and 60 min. A mosquito was considered knocked down if it laid on its side on the floor of the exposure tube and was unable to fly. At the end of the exposure period, mosquitoes were transferred into holding tubes (lined with untreated papers) by gently blowing them through the open space between the exposure tube and the holding tubes. Cotton soaked in 10 % sugar was placed on top of the holding tube. The mortality was scored 24 h post-exposure. The susceptibility status was evaluated based on the WHO criteria, i.e. a 98–100 % mortality indicated susceptibility; 90–97 % mortality required confirmation, and mortality below 90 % indicated resistance [33]. When the control mortality between 5 % and 20 % was recorded, the mean observed mortality was corrected using Abbott's method [34]. Surviving and dead mosquitoes were analyzed for species identification and knockdown resistance status (presence of L1014S *kdr*E alleles) using TaqMan assay as described previously [35].

#### 2.13.4 Social perspectives investigation

A multi-sited intrinsic case study approach was adopted to explore community perceptions and their experiences of living with the 3D-WDS, as well as the experiences of installers involved in installing the 3D-WDS in houses. This method was chosen because of its capacity to delve into individual and community living experiences in detail, to determine how these perceptions and experiences changed over time, both before and after the installation of the 3D-WDS. A purposeful sampling approach was employed to select three clusters out of seven intervention clusters that were offered 3D-WDS. Community members from the study clusters were invited to participate, and a convenience sampling method was used to select those who were present at home. Three focus groups were formed, the first focus group with male members from the study clusters (3 male focus groups from 3 clusters), the second focus group with female members from the study clusters (3 female focus groups from 3 clusters), resulting in a total of 6 FGDs across 3 study clusters. Additionally, all 7 head installers from their respective study clusters were invited to form a focus group. A total of 7 FGDs were conducted. All interviews were recorded using a digital voice recorder (Sony ES404) and the recorded interview audios were transcribed verbatim and read thoroughly several times and coded using a deductive approach to data analysis, whereby codes were influenced by research questions [36]. Thematic content analysis was applied to categorize and code the interview transcripts, whereby utterances of one participant were compared to another and later across clusters to identify common themes [[37], [38], [39]]. From each common theme, perceptions and experiences reported by all participants were specifically examined to allow formulation of patterns including main theme and sub-themes. Additionally, approximately half of the residences that were offered 3D-WDS across all 7 intervention clusters were chosen randomly for interviews with the head of the household. Interview questions were structured and organised to address topics such as common household practices to manage mosquitoes, 3D-WDS design aesthetics and durability, ease of handling and maintenance, integrity over time, installation quality, choice of materials and any additional recommendations. Interview responses were captured using ODK forms uploaded on Android tablets.

#### 2.13.5 Data management

Trained field workers used standardised data entry forms on an Android tablet (Samsung) with the Open Data Kit (ODK) Collect app to input data, which was then directly sent to a secure cloud server. The study coordinator was responsible for ensuring data validation and routine monitoring to identify any incomplete, missing, inconsistent or inaccurate data, including typos and technical errors. The project PIs closely monitored the data collection and processing through the electronic server, while the field workers had no access to the collected data. In case of technical difficulties with the electronic devices in the field, a hard copy of the respective electronic form was provided to the field workers. The data was entered into the computer with only codes of households and no names, so that they were not identifiable by the data entry clerks. The study did not and will not reveal the identity of individual participants in any reports or publications. The electronic servers, where the data was stored, were password-protected, with access granted only to the study coordinator, data management staff and project PIs. Hardcopies of data, protocol, SOPs and other documentations were kept in locked filing cabinets and only accessible to authorised study personnel. The documents will be held for at least 10 years at the host institution.

### 2.14 Statistical methods

#### 2.14.1 Outcome variables

Intervention epidemiological outcomes for the at-risk children will be assessed for one primary outcome and one secondary outcome at the level of individual health. The primary outcome will be malaria prevalence, a binary variable measured as the mRDT test result. The secondary outcome will be haemoglobin level, a strong correlate of recurring malarial episodes, as a continuous variable measured as concentration g/dl using the HemoCue analyser.

To examine whether 3D-WDS could influence entomological parameters, we will test for intervention effects on indoor mosquito densities, measured as the number of mosquitoes captured in CDC-LTs per night, and the entomological inoculation rate (EIR), measured as the number of infective bites per person per unit time, both as secondary outcomes.

#### 2.14.2 Baseline patterns

We will investigate the baseline surveys to understand two different patterns [1]: whether any demographic or health variables differed systematically between control and intervention clusters [2]; which demographic and geographic variables were associated with the outcome variables, before any intervention was made. The following variables will be compared between control and intervention clusters: age, gender, malaria prevalence, haemoglobin levels, indoor mosquito densities and EIR. These variables will be tested in a (generalized) linear mixed model with the demographic/health variables as the response variables and cluster type as the only predictor. For the baseline models for patient outcomes, the following variables will be examined as predictors: age, gender and distance to nearest health centre. These three variables will be fitted in a single model, which will be then simplified stepwise by removing any predictors with p > 0.05 until only significant predictors remain.

#### 2.14.3 Intervention effects

Intervention effects on patient outcomes will be assessed in a multivariate model that also includes the baseline variables specified above, including controlling for age and monthly precipitation squared. These intervention models will use data from all post-intervention surveys and will include time since baseline as a predictor variable, to allow for potential gradual or cumulative effects over the course of follow-up. Intervention/control will be included in these models as a main effect and also in interaction with time, allowing outcomes to change through time in one group but not in the other (e.g. a gradual decrease in malaria prevalence in intervention clusters vs. no change in control clusters). If the interaction will not be significant, it will be removed from the model and the main effect of intervention assessed.

In addition, we will test for any overall or intervention-driven change from baseline to post-intervention by pooling all post-intervention surveys and testing for an interaction between intervention. Due to the distribution of bed nets as part of the protocol, it will be possible that all participating households would experience a reduction in epidemiological or entomological outcomes, but that the reduction would be even bigger for households receiving 3D-WDS intervention.

#### 2.14.4 Model structure

All statistical modelling will be done with mixed-effects models, using the lme4 package in R [40]. All models of patient outcomes will include a random effect (random intercept) of household nested within cluster. This will allow for non-independence of sampling units by allowing the response variable to vary between clusters and in addition between households, not in association to any of the modelled fixed effects but in relation to any unobserved sources of structured error. Child ID will not be included as a random effect because the spontaneous nature of which children each household presented for examination at each visit meant that few children will be resampled often enough to be able to informatively model the individual-level variation. Baseline models will be fitted using only cluster ID as the random effect, due to limited sample sizes within each household. Malaria prevalence as measured by mRDT will be modelled in a binomial model with a logit link, and haemoglobin level will be modelled in a Gaussian model with an identity link.

Indoor mosquito densities and EIR will be analyzed at the level of clusters, using total counts of 24-night CDC-LTs collections per cluster in every cross-sectional survey. Hence, the models will include only a random effect (random intercept) of cluster ID. Count data will be modelled in a Poisson model with a log link; potential overdispersion will be accounted for by including an observation-level random effect. EIR will be modelled in a binomial model with a logit link weighted by the number of mosquitoes caught in each sample.

Community members’ perceptions will be qualitatively analyzed using data from questionnaires, focus group discussions and in-depth interviews. Additionally, quantitative data from the household questionnaire survey will be summarised using appropriate descriptive statistics.

### 2.15 Monitoring

The University of Helsinki served as the trial sponsor and primary coordinating centre, overseen by the chief investigator and co-investigators. Meanwhile, NIMR, AMRC functioned as the local coordinating centre, comprising local investigators, an experienced management team, field entomologists and laboratory technicians. A co-investigator from the primary coordinating centre, along with local co-investigators, directed trial activities, implemented the field study protocol, managed day-to-day operations under the chief investigator's supervision, and ensured that data collection adhered to standard operating procedures (SOPs). SOPs were developed based on Good Clinical Practice (GCP) and Good Field Entomology Practice (GFEP). Study investigators held weekly meetings to assess trial progress, while a Trial Steering Committee (TSC), consisting of key researchers from Finland and Tanzania, including the principal investigator (PI) and co-PIs, met remotely after each survey to oversee data collection and review overall trial progress.

## 3 Discussion

Efforts to manage malaria in sub-Saharan Africa over the last two decades have successfully averted numerous clinical cases. The widespread use of LLINs and IRS has been pivotal in reducing malaria-related deaths. However, the ongoing rise in insecticide-resistant mosquitoes and drug-resistant parasites poses a threat to the sustained effectiveness of these control measures. There is a clear imperative to invest in the research and implementation of alternative vector control strategies that do not rely on insecticides, are environmentally friendly, sustainable and socially acceptable.

The 3D-Screen offers a non-insecticidal alternative that addresses the urgent demand for novel vector control methods. These screens can be easily incorporated into existing window frames, requiring minimal structural adjustments. This provides a durable and effective complement to existing vector control measures including LLINs, IRS, coils and other repellents, without the need for their discontinuation or replacement.

The project has been designed to test whether using 3D-WDS in windows of houses, in addition to LLINs, provides additional protection against malaria mosquitoes through a large-scale cRCT in the selected communities of Muheza, Tanzania. The primary outcomes of the study will provide information about the efficacy of screening windows with 3D-WDS and trapping host-seeking malaria vectors on malaria prevalence, indoor mosquito densities and EIR. Baseline data collection will enable comparisons before and after the intervention, while separating intervention clusters by sufficient distances (≥2 km) will minimize potential spill-over effects, ensuring an effective evaluation of the impact of 3D-WDS. With extensive coverage (>80 %) of 3D-WDS in the intervention clusters, baseline measurements and a follow-up period of 50 weeks will track its gradual effectiveness. Comprehensive data collection focused on various outcomes, including malaria prevalence, indoor vector densities, EIR and community acceptance. This approach will provide a thorough understanding of the 3D-WDS's impact from multiple perspectives. Insights gained will be crucial for optimizing future scaling-up efforts and informing improvements for more effective designs, even if no significant effects are observed.

Creating a physical barrier between mosquitoes and hosts effectively protects against mosquito-borne diseases. Traditionally, house screening involves fitting mosquito screens to windows and doors to prevent mosquito entry. The 3D-WDS is a novel mosquito screen that not only blocks mosquito entry but also traps mosquitoes attempting to enter or escape from the house after a successful blood meal. Widespread use of 3D-WDS could therefore reduce mosquito numbers, blood-engorged mosquitoes in communities and reduce malaria transmission.

However, achieving high community coverage for such interventions remains a challenge. To address this, the study involved community members, including local carpenters and house owners in the installation process of the 3D-WDS. This approach helped to foster a sense of ownership, enhance health awareness and encourage the adoption of the new intervention. Engaging the community in these ways could ultimately contribute to reducing disease incidence and improving the effectiveness of the intervention.

## 4 Ethics and dissemination

### 4.1 Ethics

This study was conducted in complete accordance with the guidelines set by the international conference on Harmonization Tripartite Guideline for GCP, The Declaration of Helsinki, and the International Guidelines for Ethical Review of Epidemiological Studies. The study received full ethical approval from the National Health Research Ethics Review Committee (NatHREC), comprising the Medical Research Coordinating Committee (MRCC) and the Ministry of Health, Community Development, Gender, Elderly and Children, United Republic of Tanzania; certificate number NIMR/HQ/R.8c/Vol.I/1885, and Helsinki and Uusimaa Hospital District medical research ethics committee ref: 2242/2021. Before initiating the study, community consent was obtained both verbally and in writing from participating households. A community sensitization was conducted with a broader objective to provide awareness on malaria and mosquito control. 3D-WDS were introduced to the community, and their potential benefits, installation and other practical matters were thoroughly explained. Community leaders of participating hamlets, District medical and public health officers and health workers were invited to explain the clinical and entomological procedures. All questionnaires, surveys and meetings were conducted in Kiswahili.

### 4.2 Confidentiality

All procedures for data collection, management, storage and manipulation adhered to the SOPs to ensure consistency and accuracy. Participant confidentiality was safeguarded by keeping paper case report forms (CRFs) containing personal information in a secured cabinet accessible only by authorised personnel. The unique identifier was used during analysis to maintain anonymity.

### 4.3 Dissemination plans

All trial findings will be communicated with international policymakers such as the WHO Vector Control Advisory Group (VCAG), the malaria policy advisory group (MPAC), and the WHO prequalification team for vector control products to promote the adoption of sustainable vector control methods. The project also engaged national, local, and community authorities and leaders. Meetings were held midway and at the end of the follow-up to update village and community leaders on progress and facilitate dissemination of findings within the community. All publications and findings arising from this trial will be disseminated as open access.

### Availability of data and materials

Anonymised data from the final trial will be made available upon reasonable request following the publication of the study results.

## CRediT authorship contribution statement

**William N. Kisinza:** Writing – review & editing, Supervision, Resources, Project administration, Conceptualization. **Subam Kathet:** Writing – original draft, Supervision, Project administration, Investigation, Formal analysis, Data curation. **Victor Mwingira:** Writing – review & editing, Supervision, Resources, Project administration. **Maija Meri:** Writing – review & editing, Investigation. **Frank S. Magogo:** Writing – review & editing, Supervision, Project administration. **Veneranda M. Bwana:** Writing – review & editing, Supervision, Project administration, Methodology. **Hanna Granroth-Wilding:** Writing – review & editing, Formal analysis. **Pendael Machafuko:** Writing – review & editing, Project administration. **Patrick Tungu:** Writing – review & editing, Methodology. **Mikko Aalto:** Writing – review & editing, Conceptualization. **Tomi Hakala:** Writing – review & editing, Resources, Methodology, Conceptualization. **Markku Honkala:** Resources, Methodology, Conceptualization. **Seppo Meri:** Writing – review & editing, Supervision, Resources, Project administration, Funding acquisition, Conceptualization. **Ayman Khattab:** Writing – review & editing, Writing – original draft, Supervision, Resources, Project administration, Funding acquisition, Formal analysis, Conceptualization.

## Ethics statement

Ethical approval was obtained from the National Health Research Ethics Review Committee, Tanzania (NIMR/HQ/R.8c/Vol.I/1885), and the Helsinki and Uusimaa Hospital District Medical Research Ethics Committee (Ref: 2242/2021).

## Funding

The study was funded through the financial support from Jane ja Aatos Erkon Säätiö (Grant no. 4244-67694).

## Declaration of competing interest

The authors declare that they have no known competing financial interests or personal relationships that could have appeared to influence the work reported in this paper.

## List of abbreviations

**3D-WDS**: Three-Dimensional Window Screen
**AMRC**: Amani Medical Research Centre
**ALu**: Artemether-Lumefantrine
**CDC**: Centre for Disease Control and Prevention
**CRF**: Case Report form
**cRCT**: Cluster Randomised Controlled Trial
**DBS**: Dried blood Spot
**EIR**: Entomological Inoculation Rate
**FGDs**: focus group discussions
**GCP**: Good Clinical Practice
**GFEP**: Good Field Entomology Practice
**GLP**: Good Laboratory Practice
**Hb**: Haemoglobin
**IRB**: Institutional Review Board
**IRS**: Indoor Residual Spraying
***kdr*-E**: Knock Down Resistance-East
**ISRCTN**: International Standard Randomised Controlled Trial Number
**ITNs**: Insecticide Treated Nets
**LLINs**: Long-lasting Insecticide Treated Nets
**LT**: Light Trap
**mRDT**: Malaria Rapid Diagnostic Test
**MRCC**: Medical Research Coordinating Committee
**MPAC**: Malaria Policy Advisory Group
**NatHREC**: National Health Research Ethics Review Committee
**NIMR**: National Institute for Medical Research
**NMCP**: National Malaria Control Programme
**PCR**: Polymerase chain reaction
**RCT**: Randomised Controlled Trial
**RT-PCR**: Real Time Polymeric Chain Reaction
**SOP**: Standard Operating Procedure
**TSC**: Trial Steering Committee
**VCAG**: Vector Control Advisory Guidelines
**WHO**: World Health Organization
